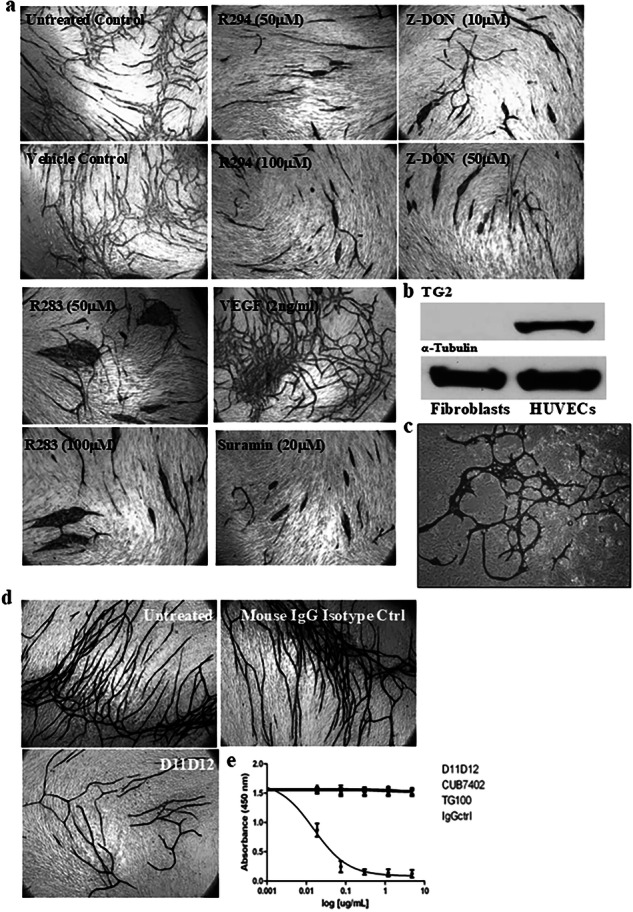# Correction: A novel extracellular role for tissue transglutaminase in matrix-bound VGF-mediated angiogenesis

**DOI:** 10.1038/s41419-026-08821-y

**Published:** 2026-05-13

**Authors:** Z. Wang, M. Perez, S. Caja, G. Melino, T. S. Johnson, K. Lindfors, M. Griffin

**Affiliations:** 1 School of Life and Health Sciences, Aston University, Aston Triangle, Birmingham, UK; 2 Paediatric Research Centre, University of Tampere and Tampere University Hospital, Tampere, Finland; 3 MRC Toxicity Unit, University of Leicester, Leicester, UK; 4 Academic Nephrology Unit, Sheffield Kidney Institute, School of Medicine and Biomedical Sciences, University of Sheffield, Sheffield, UK

Correction to: *Cell Death & Disease* 10.1038/cddis.2013.318, published online 19 September 2013

The following corrections are made to the article.

Figure 2d panel description now reads: Effect of TG2-neutralising antibody D11D12 on endothelial cell tubule formation in the co-culture assay. Culture medium was supplemented with either the mouse monoclonal TG2 activity neutralising antibody D11D12 (0.1 μg/ml) added to the co-culture system from day 1 (24h after seeding). Controls consisted of either untreated cultures or isotype-matched IgG. After 12 days, the tubule formation was analysed as described in (Fig. 2a) (see Supplementary Table S1) Figure 2 is also updated with correct panel d.

The text is changed from “To confirm the extracellular importance and specificity of TG2 in the formation of HUVEC tubules, co-cultures were incubated with the TG2-specific transamidating inactivating monoclonal antibody D11D12. Incubation with this antibody led to a significant reduction of tubule formation (around 50%) (Figure 2d, Supplementary Table S1) and a significant reduction in extracellular TG2 activity (Figure 2e).” to “To confirm the extracellular importance and specificity of TG2 in the formation of HUVEC tubules, co-cultures were incubated with the TG2-specific transamidating neutralising monoclonal antibody D11D12. Incubation with this antibody led to a significant reduction of tubule formation (around 50%) (Fig. 2d, Supplementary Table S1) unlike its isotype control and non-treated wells with a significant reduction in extracellular TG2 activity (Fig. 2e).”

**Old Figure 2** 
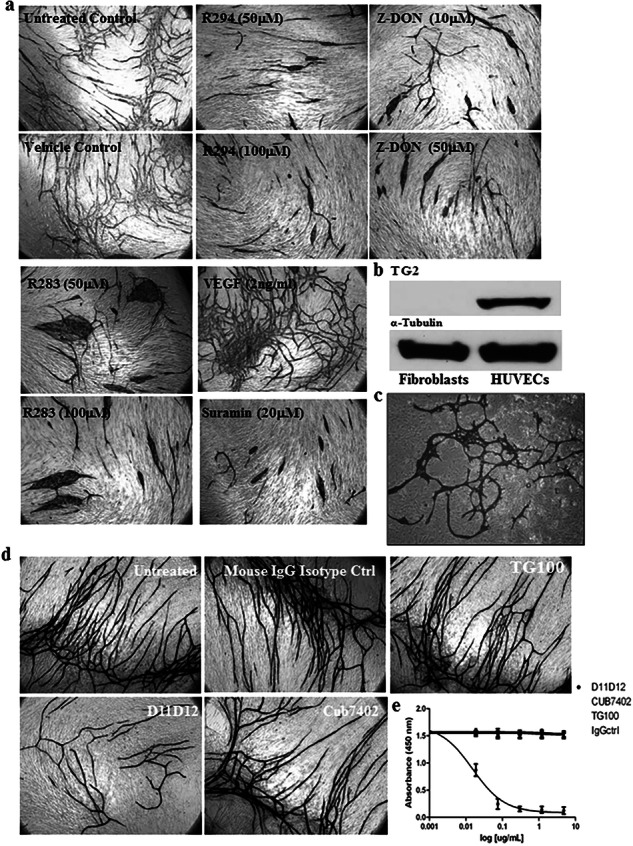


**New Figure 2**